# ICFEP Secundária Existe? Sim, mas não Deveria ser Chamada de ICFEP

**DOI:** 10.36660/abc.20240296

**Published:** 2024-10-08

**Authors:** Humberto Villacorta, Pedro Schwartzmann

**Affiliations:** 1 Universidade Federal Fluminense Niterói RJ Brasil Universidade Federal Fluminense, Niterói, RJ – Brasil; 2 Hospital Unimed Ribeirão Preto e Centro de Pesquisa CAPED Ribeirão Preto SP Brasil Hospital Unimed Ribeirão Preto e Centro de Pesquisa CAPED, Ribeirão Preto, SP – Brasil

**Keywords:** Insuficiência Cardíaca, Insuficiência Cardíaca Diastólica, Terminologia

## Introdução

A insuficiência cardíaca com fração de ejeção preservada (ICFEP) é definida como uma síndrome com sintomas e sinais de insuficiência cardíaca (IC) e fração de ejeção do ventrículo esquerdo (FEVE) ≥ 50%.^1^ Essa definição simplista pode levar a erros de diagnóstico por confundir doenças não cardíacas com ICFEP. A definição de ICFEP disponível pela Sociedade Europeia de Cardiologia (ESC) é mais completa e requer a presença de sintomas e sinais de IC com evidências de anomalias cardíacas estruturais e/ou funcionais e/ou peptídeos natriuréticos elevados e FEVE ≥ 50%.^2^

A fisiopatologia da ICFEP não é totalmente compreendida. Entretanto, é basicamente considerada uma síndrome heterogênea e sistêmica, associada à inflamação, envelhecimento, predisposição genética e múltiplas comorbidades, como hipertensão, diabetes mellitus, obesidade e fibrilação atrial. A doença progride para perda do relaxamento ventricular esquerdo, remodelação atrial esquerda e sinais de congestão.^1^

O quadro descrito acima não inclui doenças secundárias. Entretanto, algumas doenças cardíacas específicas e até mesmo anormalidades não cardíacas podem, às vezes, apresentar sinais de congestão e FEVE ≥ 50%, mas não são verdadeiramente ICFEP. Esta situação é denominada por alguns autores como ICFEP "secundária".^3,4^ Neste artigo, apresentamos argumentos que são contra denominar essa situação de ICFEP "secundária". Portanto, evitaremos o termo ICFEP "primária" e, em vez disso, utilizaremos o termo ICFEP "verdadeira".

### O que dizem as Diretrizes?

As principais Diretrizes e Consensos sobre IC sempre abordam o manejo da ICFEP.^2,5,6^ O diagnóstico e as recomendações de tratamento constantes nestas Diretrizes são voltados principalmente à ICFEP verdadeira, uma doença primária do miocárdio sem uma etiologia específica. Conforme esperado, as diretrizes mencionam uma situação em que doenças específicas podem mimetizar a ICFEP, mas não a consideram uma ICFEP verdadeira. Além disso, a terminologia "mimetizar", que tem sido utilizada em diretrizes e outras publicações, reforça o conceito de que não há uma definição universalmente aceita da terminologia para a ICFEP secundária. A verdadeira mensagem por trás da palavra "mimetizar" é: "Tenha cuidado. O seu diagnóstico de ICFEP pode estar incorreto. Descarte doenças que mimetizam a ICFEP, mas que não são a ICFEP verdadeira".

A Atualização de Tópicos Emergentes de 2021 da Diretriz Brasileira de Insuficiência Cardíaca^5^ afirma o seguinte no capítulo sobre ICFEP: "É fundamental investigar condições reversíveis que podem estar associadas à ICFEP ‘secundária’, como cardiomiopatias infiltrativas e restritivas, além de causas alternativas de intolerância ao esforço". Observa-se que a palavra "secundária" está entre aspas, indicando que os autores não adotam essa nomenclatura.

As Diretrizes da ESC de 2021 sobre IC^2^ afirmam que: "É importante excluir outras condições que podem mimetizar a síndrome ICFEP (por exemplo, doença pulmonar, anemia, obesidade e descondicionamento)". No Consenso de 2019 sobre ICFEP da Heart Failure Association (HFA) da ESC,^7^ há bastante clareza sobre esta questão: "É preciso excluir os "mimetizadores" de ICFEP, como doença da válvula cardíaca, arritmias e constrição pericárdica. Da mesma forma, um paciente com FEVE normal e sintomas semelhantes aos da IC causados por doença arterial coronariana significativa também não é considerado portador de ICFEP".

E, por fim, o Consenso de 2023 do ACC sobre ICFEP^6^ afirma que "…um indivíduo com evidência de congestão e FE preservada pode apresentar ‘IC atribuída a cardiomiopatias especiais’ ou incomuns, como cardiomiopatia infiltrativa/restritiva, cardiomiopatia hipertrófica, doença cardíaca valvular ou doença pericárdica; ou, se nenhuma dessas condições for sugerida de acordo com a apresentação clínica ou exame de diagnóstico, então o diagnóstico de ICFEP é estabelecido por exclusão…". A mensagem aqui indica que, antes de confirmar o diagnóstico de ICFEP, é preciso descartar as condições que mimetizam a ICFEP.

### O que exatamente significa a terminologia "mimetizador" de ICFEP?

Conforme mencionado acima, diversas doenças específicas podem apresentar sintomas semelhantes aos da IC – e, às vezes, congestão real – e uma FEVE ≥ 50%, mas, em última análise, não são consideradas a ICFEP verdadeira. Essas anormalidades são geralmente chamadas de "mimetizadores" ou "mascaradores" de ICFEP. Elas podem ser distúrbios cardíacos ou sistêmicos e têm tratamento específico. Podem ser confundidas com a ICFEP, levando a erros no plano de tratamento caso não sejam detectadas.

**Figura 1:**
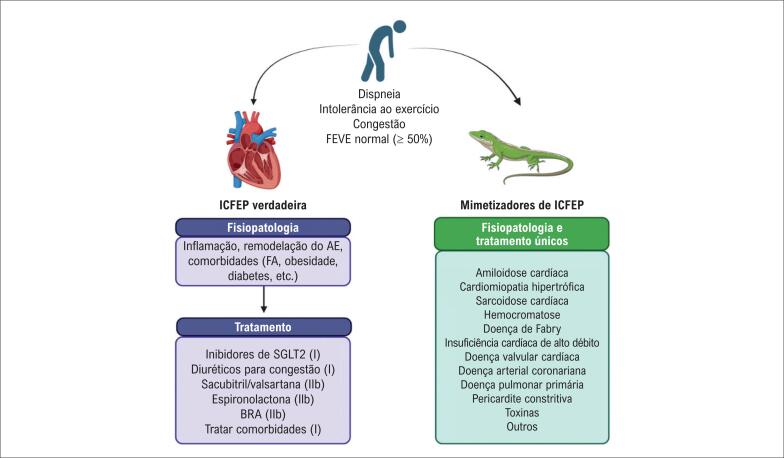
Fisiopatologia e tratamento de pacientes com insuficiência cardíaca com fração de ejeção preservada (ICFEP) e daqueles com doenças que mimetizam a ICFEP. Cada uma das doenças do último grupo possui um tratamento específico e único, diferentemente do tratamento da ICFEP verdadeira. Entre parênteses estão as classes de recomendação de acordo com as diretrizes sobre IC.^2,5,6^

Alguns exemplos são as cardiomiopatias infiltrativas e restritivas, como a amiloidose e a doença de Fabry.^8^ A fisiopatologia e os tratamentos dessas doenças são completamente diferentes daqueles da ICFEP verdadeira. A amiloidose é uma doença sistêmica que pode afetar o coração e é causada pela deposição de substância amiloide no miocárdio. O tratamento específico é baseado em medicamentos como o tafamidis.^8^ A doença de Fabry é causada por um erro genético que leva à deficiência da enzima alfa-galactosidase. O tratamento é a reposição dessa enzima ou a estabilização com chaperonas.^8^ Da mesma forma, muitos outros distúrbios, como pericardite constritiva, doença valvar cardíaca, doença cardíaca coronariana etc., têm tratamentos específicos que são completamente diferentes daquele da ICFEP verdadeira, conforme demonstrado na Figura 1.

### Por que não deve ser chamada de ICFEP?

Primeiro, como explicado anteriormente, os mimetizadores de ICFEP possuem fisiopatologias única e tratamentos específicos e, portanto, não devem ser considerados como ICFEP verdadeira. Em segundo lugar, também é importante não se referir a tais doenças como ICFEP, pois pode causar mal-entendidos na comunidade médica e nos estudantes de medicina. A ICFEP em si é uma síndrome complexa. Incluir, na definição de ICFEP, doenças que a mimetizam, mas que não são a ICFEP verdadeira, pode causar equívocos. Para elucidar com um exemplo, um estudante de medicina sugeriu prescrever inibidores de SGLT2 a um paciente com estenose aórtica grave com FEVE preservada porque havia lido que esse é o único tratamento médico aprovado para ICFEP. Acontece que pacientes com estenose aórtica foram excluídos dos ensaios realizados sobre inibidores de SGLT2 na ICFEP, e o tratamento definitivo envolve cirurgia cardíaca ou implante de válvula percutânea.

Gostaríamos de relembrar um caso recente em que um termo não adequado causou mal-entendido. Você se lembra do termo "IC com FE de médio alcance" (ICFEm, FE 40-49%)? A ESC criou este termo em 2016 para estimular debates e pesquisas sobre essa variedade de FE, uma vez que esses pacientes eram frequentemente excluídos dos ensaios clínicos.^9^ No entanto, a comunidade médica interpretou como se uma nova classe tivesse sido criada, completamente diferente de ICFER e ICFEP. A ESC, por fim, corrigiu o termo em 2021 para refletir o que ele realmente significa: IC com FE levemente reduzida, esclarecendo que é um continuum dentro do espectro de IC.^2^

## Conclusão

Concluindo, a definição e a compreensão da ICFEP são caracterizadas por nuances e estão evoluindo. Apesar dos esforços para refinar a definição, persistem desafios em distinguir a ICFEP verdadeira das condições que mimetizam sua apresentação. Essas mimetizações incluem diversos distúrbios cardíacos e sistêmicos e cada um exige estratégias de tratamento distintas. É importante ressaltar que considerar erroneamente essas condições como ICFEP pode levar a abordagens de tratamento incorretas e gerar mal-entendidos na comunidade médica.

Portanto, é fundamental manter a clareza na terminologia e reconhecer a fisiopatologia única e as considerações terapêuticas dos mimetizadores de ICFEP. Ao aderir a critérios de diagnóstico abrangentes e ter cautela para excluir condições mascaradoras, os médicos podem otimizar o atendimento ao paciente e mitigar o risco de um manejo incorreto dessa síndrome complexa.
