# Errata

**DOI:** 10.36660/abc.20250119

**Published:** 2025-03-10

Edição de Setembro de 2024, vol. 121(9): e20240296

No Ponto de Vista “ICFEP Secundária Existe? Sim, mas não Deveria ser Chamada de ICFEP”, com número de DOI: https://doi.org/10.36660/abc.20240296, publicado no periódico Arquivos Brasileiros de Cardiologia, Arq Bras Cardiol. 2024; 121(9):e20240296, na página 2, alterar a frase: Você se lembra do termo “IC com FE de médio alcance” (ICFEm, FE 40-49%)? por: Você se lembra do termo “IC com FE intermediária” (ICFEI, FE 40-49%)?

