# Supplementary material for: ICFEP Secundária Existe? Sim, mas não Deveria ser Chamada de ICFEP
Source: Arq Bras Cardiol. 2024 Oct 8;121(9):e20240296. [Article in Portuguese] doi: 10.36660/abc.20240296 (PMC12092047; doi:10.36660/abc.20240296)
Supplement: Translation

# Translation: Does Secondary HFpEF Exist? Yes, but it Should not be Called HFpEF

**DOI:** 10.36660/abc.20240296i

**Authors:** Humberto Villacorta, Pedro Schwartzmann

**Affiliations:** 1 Niterói RJ Brazil Universidade Federal Fluminense, Niterói, RJ – Brazil; 2 Ribeirão Preto SP Brazil Hospital Unimed Ribeirão Preto e Centro de Pesquisa CAPED, Ribeirão Preto, SP – Brazil

**Keywords:** Heart Failure, Diastolic Heart Failure, Terminology

## Introduction

Heart failure with preserved ejection fraction (HFpEF) is defined as a syndrome with symptoms and signs of heart failure (HF) and left ventricular ejection fraction (LVEF) ≥ 50%.^1^ This simplistic definition may lead to diagnostic errors by mistaking non-cardiac diseases for HFpEF. The European Society of Cardiology (ESC) offers a more comprehensive definition HFpEF, which requires the presence of symptoms and signs of HF, along with evidence of structural and/or functional cardiac abnormalities, and/or elevated natriuretic peptides, with an LVEF ≥ 50%.^2^

The pathophysiology of HFpEF is not fully understood. Still, it is generally considered a heterogenous and systemic syndrome associated with inflammation, aging, genetic predisposition, and multiple comorbidities, such as hypertension, diabetes mellitus, obesity, and atrial fibrillation. The disease ultimately progresses to loss of left ventricular relaxation, left atrial remodeling, and signs of congestion.^1^

The condition described above does not include secondary diseases. However, some specific cardiac diseases and even non-cardiac abnormalities may occasionally present with signs of congestion and LVEF ≥ 50% but are not true HFpEF. Some authors refer to this situation as "secondary" HFpEF.^3,4^ In this article, we aim to present the arguments against labeling this situation as "secondary" HFpEF. Consequently, we will avoid using the term "primary" HFpEF and instead refer to it as "true" HFpEF.

### What do the Guidelines say?

The main Guidelines and Consensus on HF always address the management of HFpEF.^2,5,6^ The diagnosis workup and treatment recommendations in these Guidelines are mainly focused on the true HFpEF, a primary myocardial disease without a specific underlying etiology. As expected, they mention a situation where specific diseases may mimic HFpEF but do not consider these cases to be true HFpEF. Moreover, the use of the term "mimics," which has been used in guidelines and other publications, reinforces the concept that there is no universally accepted definition for the secondary HFpEF terminology. The true message behind the word "mimics" is, "Be careful. Your diagnosis of HFpEF may be incorrect. Rule out diseases that mimic HFpEF but are not true HFpEF."

The 2021 Emerging Topics Update of the Brazilian Heart Failure Guideline^5^ states the following in the HFpEF chapter: "It is essential to investigate potentially reversible conditions associated with ‘secondary’ HFpEF, such as infiltrative and restrictive cardiomyopathies, and additionally consider alternative causes of exercise intolerance." Notably, the word "secondary" is in quotation marks, indicating that the authors do not adopt this nomenclature.

The 2021 ESC Guidelines on HF^2^ state: "It is important to exclude other conditions that might mimic the HFpEF syndrome (e.g., lung disease, anemia, obesity, and deconditioning)." In the 2019 Consensus of HFpEF by the Heart Failure Association (HFA) of the ESC,^7^ they make this issue crystal clear: "Heart failure with preserved ejection fraction ‘masqueraders’ such as heart valve disease, arrhythmias, and pericardial constriction need to be excluded. Similarly, a patient with normal LVEF and HF-like symptoms caused by significant coronary artery disease is also not considered to have HFpEF."

Finally, the 2023 ACC Consensus on HFpEF^6^ states that "…an individual with evidence of congestion and preserved EF may have ‘HF attributed to special’ or unusual cardiomyopathies such as infiltrative/restrictive cardiomyopathy, hypertrophic cardiomyopathy, valvular heart disease, or pericardial disease; or, if the clinical presentation or diagnostic testing suggests no such condition, then a diagnosis of HFpEF is established by exclusion…". The message here is that before confirming the diagnosis of HFpEF, one must rule out conditions that mimic HFpEF.

### What exactly does the HFpEF "mimic" terminology stand for?

As mentioned above, several specific diseases may present with HF-like symptoms – and sometimes true congestion – and an LVEF ≥ 50% but are ultimately not considered true HFpEF. These abnormalities are usually called HFpEF "mimics" or "masqueraders." They may be cardiac or systemic disorders, each with specific treatments. If not suspect, they may be misidentified as HFpEF, thus leading to errors in the treatment plan.

Examples include infiltrative and restrictive cardiomyopathies, such as amyloidosis and Fabry disease.^8^ The pathophysiology and treatment of these diseases are completely different from that of true HFpEF. Amyloidosis is a systemic disease that may affect the heart due to the deposition of amyloid substances in the myocardium. The specific treatment is based on drugs such as tafamidis.^8^ Fabry disease results from a genetic error that leads to a deficiency of the enzyme alpha-galactosidase. The treatment is the reposition of such enzyme or the stabilization with chaperones.^8^ Likewise, many other disorders, such as constrictive pericarditis, valvular heart disease, coronary heart disease, etc., have specific treatments that are completely different from those of true HFpEF, as shown in Figure 1.

**Figure 1:** Pathophysiology and treatment of patients with heart failure with preserved ejection fraction (HFpEF) and those with diseases that mimic HFpEF. Each of the diseases in the latter group has a specific and unique treatment as opposed to the treatment of true HFpEF. In parentheses are the recommended classes according to the HF guidelines.^2,5,6^

### Why should it not be called HFpEF?

First, as previously explained, HFpEF mimics have unique pathophysiologies and specific treatments and, therefore, should not be seen as true HFpEF. Second, another important reason for not referring to such diseases as HFpEF is that it may cause confusion in the medical community and medical students. HFpEF itself is a complex syndrome. Including diseases that mimic HFpEF but are not true HFpEF in its definition causes lead to misunderstandings. For instance, one medical student suggested prescribing SGLT2 inhibitors to a patient with severe aortic stenosis with preserved LVEF because he had read that this is the only medical treatment approved for HFpEF. It turns out that patients with aortic stenosis were excluded from the trials of SGLT2 inhibitors in HFpEF, and their ultimate treatment is heart surgery or percutaneous valve implantation.

We would like to recall a recent case where a misplaced term caused confusion. Do you remember the term "HF with mid-range EF" (HFmrEF, EF 40-49%)? The ESC created this term in 2016 to stimulate debate and research in this range of EF, as these patients were often excluded from clinical trials.^9^ However, the medical community misinterpreted it as the creation of a new class, completely different from HFrEF and HFpEF. The ESC eventually corrected the term in 2021 to what it really is – HF with mildly reduced EF, clarifying that it is a continuum within the HF spectrum.^2^

## Conclusion

In conclusion, the definition and understanding of HFpEF are both nuanced and evolving. Despite efforts to refine the definition, challenges persist in distinguishing true HFpEF from conditions that mimic its presentation. These mimics encompass a diverse array of cardiac and systemic disorders, each requiring distinct management strategies. Importantly, mislabeling these conditions as HFpEF can lead to incorrect treatment approaches and create confusion within the medical community.

Therefore, it is imperative to maintain clarity in terminology and recognize the unique pathophysiology and therapeutic considerations of HFpEF mimics. By adhering to comprehensive diagnostic criteria and remaining vigilant in excluding masquerading conditions, clinicians can optimize patient care and mitigate the risk of mismanagement in this complex syndrome.
